# Deep learning-based diagnosis from endobronchial ultrasonography images of pulmonary lesions

**DOI:** 10.1038/s41598-022-17976-5

**Published:** 2022-08-12

**Authors:** Takamasa Hotta, Noriaki Kurimoto, Yohei Shiratsuki, Yoshihiro Amano, Megumi Hamaguchi, Akari Tanino, Yukari Tsubata, Takeshi Isobe

**Affiliations:** Department of Internal Medicine, Division of Medical Oncology and Respiratory Medicine, Shimane University, 89-1 Enya-cho, Izumo, Shimane 693-8501 Japan

**Keywords:** Translational research, Lung cancer, Biomedical engineering

## Abstract

Endobronchial ultrasonography with a guide sheath (EBUS-GS) improves the accuracy of bronchoscopy. The possibility of differentiating benign from malignant lesions based on EBUS findings may be useful in making the correct diagnosis. The convolutional neural network (CNN) model investigated whether benign or malignant (lung cancer) lesions could be predicted based on EBUS findings. This was an observational, single-center cohort study. Using medical records, patients were divided into benign and malignant groups. We acquired EBUS data for 213 participants. A total of 2,421,360 images were extracted from the learning dataset. We trained and externally validated a CNN algorithm to predict benign or malignant lung lesions. Test was performed using 26,674 images. The dataset was interpreted by four bronchoscopists. The accuracy, sensitivity, specificity, positive predictive value (PPV), and negative predictive value (NPV) of the CNN model for distinguishing benign and malignant lesions were 83.4%, 95.3%, 53.6%, 83.8%, and 82.0%, respectively. For the four bronchoscopists, the accuracy rate was 68.4%, sensitivity was 80%, specificity was 39.6%, PPV was 76.8%, and NPV was 44.2%. The developed EBUS-computer-aided diagnosis system is expected to read EBUS findings that are difficult for clinicians to judge with precision and help differentiate between benign lesions and lung cancers.

## Introduction

When lung cancer is suspected on chest X-ray or computed tomography examination, it is important to accurately collect cells and tissues from the suspected site to reach a definitive diagnosis. Endobronchial ultrasonography using a guide sheath (EBUS-GS) is a common technique implemented for obtaining biopsy specimens from peripheral pulmonary lesions^1^. The diagnostic yield is reported to be 83–87% when the probe is at the center of the lesion (within) and much lower at 42–61% when the probe is adjacent to the lesion^1–3^.

Generally, when performing EBUS-GS for diagnosis of a lung lesion, the suspicion of malignancy is high, and cancer diagnosis should not be missed (false negatives). When performing EBUS-GS for a peripheral lung lesion, because the technique is not performed under direct vision, it cannot be confirmed whether the biopsy was performed at the correct location.

Previous reports on ultrasound findings have classified internal tumor echo findings into three types (six subclasses) and stated that it is possible to discriminate between benign and malignant peripheral pulmonary lesions with high probability^4^. Thus, EBUS findings are very important for assisting final diagnosis. However, classification based on ultrasound findings has not been effectively utilized owing to variations in technology acquisition and accuracy.

Regarding the use of artificial intelligence (AI) in ultrasonic findings, deep learning application studies have been conducted on breast ultrasonography^5^ and intraductal papillary mucinous neoplasm of the pancreas^6^, and good results have been obtained. Histogram data collected from EBUS-GS images have been reported to be useful for diagnosing lung cancers as a method for quantitatively evaluating EBUS images of peripheral pulmonary lesions^7^. Therefore, in the present study, we examined the efficacy and consistency of AI-assisted ultrasonic findings in distinguishing benign and malignant pulmonary lesions.

## Methods

### Patient population

For this observational retrospective cohort study, we obtained EBUS images of peripheral pulmonary lesions that were recorded between April 2017 and November 2019 at our institute. Bronchoscopy was performed in midazolam-sedated patients using a flexible bronchoscope (BF-P260F, BF-260, BF-6C260, or BF-1T260; Olympus Medical Systems, Tokyo, Japan). EBUS images were obtained using a miniature ultrasound probe (UM-S20-17S, UM-S20-20R, Olympus Medical Systems) and endoscopic ultrasound processors (Endoscopic Ultrasound Center; EU-ME1, Olympus Medical Systems). The inclusion criteria for EBUS images of malignancy were histopathologically confirmed cases of lung adenocarcinoma, squamous cell lung cancer, and small cell lung cancer, diagnosed either by surgery or bronchoscopic biopsy. The inclusion criteria for EBUS images of benign lesions were bacteriological diagnosis of histopathologically confirmed cases or the disappearance of lung shadows for a minimum of 6 months of follow-up. EBUS images of poor quality were excluded from the study due to the unclear depiction of lesions. Tumor lesions were visible on all images, and multiple images were collected for the same lesion to include different distances and angles. Lesions were selected by an experienced bronchoscopist (bronchoscopy specialist, 11 years of experience in bronchoscopy, and research experience related to EBUS imaging) to generate image datasets for deep learning models. This retrospective study was approved by the Shimane University Institutional Review Board (IRB study number: 5073). The requirement for informed consent was waived due to the retrospective nature of the study, which was approved by the Shimane University Institutional Review Board. This study was conducted in accordance with the amended Declaration of Helsinki.

### Data preprocessing

Data augmentation was used to increase the variation of the image. Previous reports have shown that these techniques are effective in improving the accuracy of recognition and classification for analysis with endoscopic ultrasonography images^8^. The dataset augmentation methods used were rotation, inversion, and enlargement. Data augmentation was applied to the training image.

### Model development

**Figure 1:**
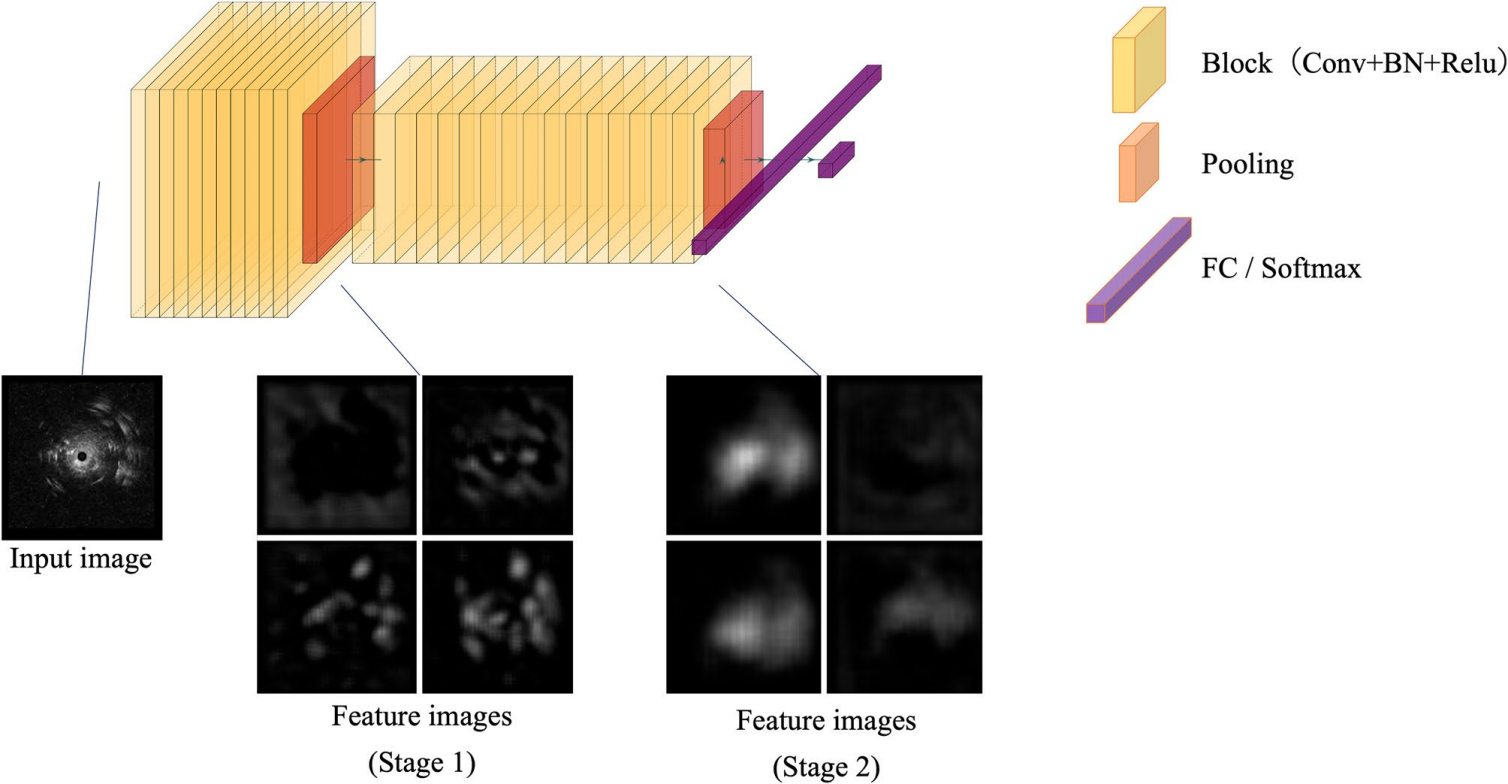
Convolutional neural network architecture in this study. Feature extraction using CNN consists of two stages, with each stage consisting of multiple blocks and one pooling layer. The first stage consists of 11 blocks and one pooling layer, while the second consists of 16 blocks and one global average pooling layer. One block consists of a convolution layer (Conv), batch normalization layer (BN), and rectified linear unit (ReLU) function. Conv is a dilated convolution. The size of the kernel is 3. Both the dilation size and padding size are 3. The number of channels of Conv in the first and second stages is 135 and 270, respectively. The classification neural network is composed of a fully connected layer (FC) and a softmax layer. The figure was generated by PlotNeuralNet and modified. PlotNeuralNet v1.0.0 (https://github.com/HarisIqbal88/PlotNeuralNet) is released under the MIT License (https://opensource.org/licenses/mit-license.php).

Our convolutional neural network (CNN) structure is shown in Fig. 1. First, the EBUS image was input to the feature extraction CNN. In the first block of the CNN, local features, such as edges and textures, were extracted from the input image. When passing through a network, the features were integrated. Finally, it was converted into a feature that was useful for discrimination between benign and malignant lesions. Next, these useful features were input into the classification neural network. In neural network classification, the probabilities of the lesion being benign or malignant are estimated. The one with the highest probability was used as the discrimination result.

### Outcome measures

**Figure 2:**
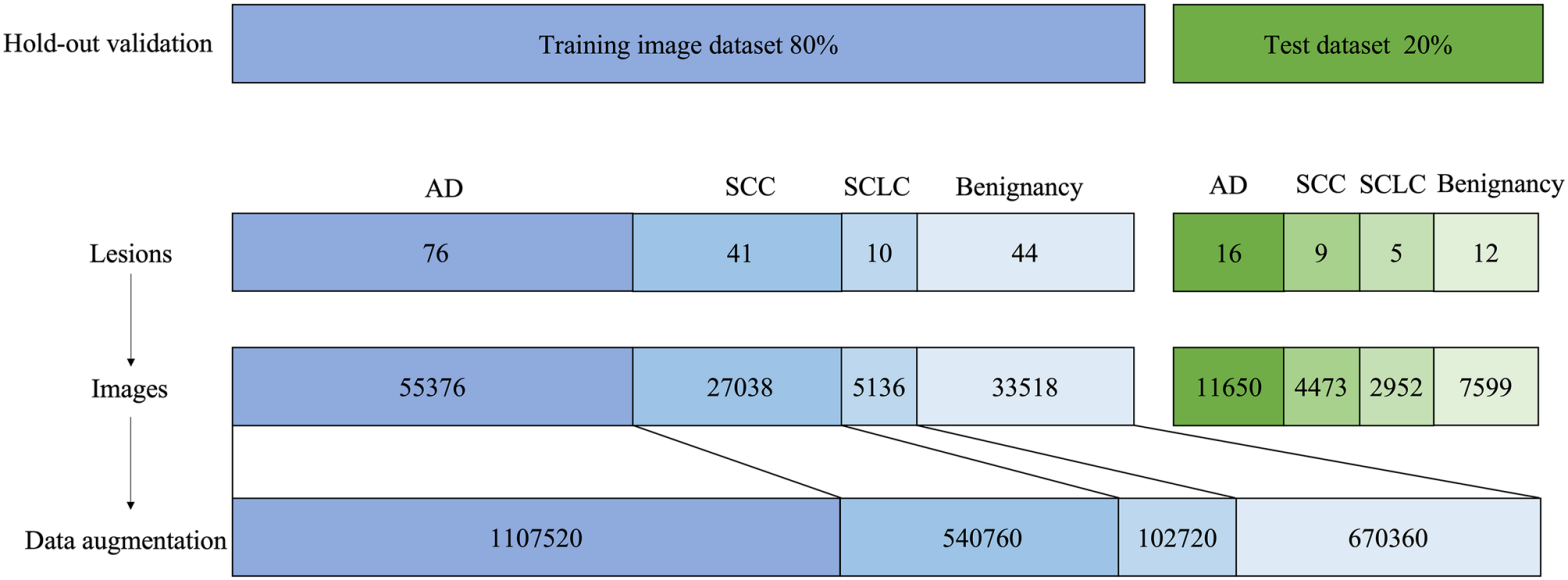
Data preprocessing flow and analysis data breakdown in this study. Training image dataset were 55,376 images of 76 adenocarcinomas, 27,038 images of 41 squamous cell carcinomas, 5136 images of 10 small cell carcinomas, and 33,518 images of 44 benign lesions. Data augmentation was applied to the training image dataset. Test dataset were 11,650 images of 16 adenocarcinomas, 4473 images of 9 squamous cell carcinomas, 2952 images of 5 small cell carcinomas, and 7599 images of 12 benign lesions. The ratio for training and test datasets was 80:20. *AD* adenocarcinoma, *SCC* squamous cell carcinomas, *SCLC* small cell lung cancer.

The entire data was divided into training data and test data to check the accuracy of the model. Using hold-out validation, the images were divided into training (80% of patients) and test sets (20% of patients) (Fig. 2). Data with a new date were used as the test sets. The classification provided by the CNN-computer-aided diagnosis (CAD) system was compared with the histopathology results. Accuracy, sensitivity, specificity, positive predictive value (PPV), and negative predictive value (NPV) were used as evaluation indexes.

To provide a comparison of the classification performance of the CNN-CAD system, four bronchoscopists were tasked with evaluating the test sets. Among them, two bronchoscopists were classified as Expert 1 (bronchoscopy specialist, 35 years of experience in bronchoscopy, research works related to EBUS imaging) and Expert 2 (bronchoscopy specialist, 7 years of experience in bronchoscopy, and research works related to EBUS imaging). The others were classified as Trainee 1 (bronchoscopy specialist, 12 years of experience in bronchoscopy) and Trainee 2 (5 years of experience in bronchoscopy) after being trained in interpreting EBUS images. The bronchoscopists received the original EBUS image without information on the CNN-CAD system classification results and provided their own classifications (benign or malignant).

### Visualization of the CNN-CAD system

CNN-based models provide excellent performance, but they lack intuitive components and are difficult to interpret. To better understand the prediction process of deep learning models, we used visualization techniques such as a novel class-discriminative localization technique and gradient-weighted class activation mapping^9^.

### Statistical analysis

Statistical analyses were performed using R (version 3.6.2, R Foundation for Statistical Computing, Vienna, Austria). Quantitative variables were reported as mean and standard deviation, and qualitative variables were reported as frequency and percentage. Categorical data were analyzed using Fisher’s exact test. Accuracy, sensitivity, specificity, PPV, and NPV between the bronchoscopists and the CNN-CAD system were expressed as percentages. The accuracy was compared using the McNemar test. Statistical significance was defined as a *P*-value < 0.05.

## Results

### Clinicopathological and patient characteristics

After applying the inclusion and exclusion criteria, we finally used 2,421,360 images of 171 peripheral pulmonary lesions as the training image dataset (55,376 images of 76 adenocarcinomas, 27,038 images of 41 squamous cell carcinomas, 5136 images of 10 small cell carcinomas, and 33,518 images of 44 benign lesions). Data augmentation was applied to the training image dataset (1,107,520 images of adenocarcinomas, 540,760 images of squamous cell carcinomas, 102,720 images of small cell carcinomas, and 670,360 images of benign lesions). Data recorded from April 2017 to June 2019 were used as the training dataset. We also procured a test dataset of 26,674 EBUS images of 42 peripheral pulmonary lesions (11,650 images of 16 adenocarcinomas, 4473 images of 9 squamous cell carcinomas, 2952 images of 5 small cell carcinomas, and 7599 images of 12 benign lesions) (Fig. 2). We then collated an independent test dataset of 42 peripheral pulmonary lesions that had been recorded from June 2019 to November 2019 (16 adenocarcinoma, 9 squamous cell carcinoma, 5 small cell carcinoma, and 12 benign lesions) (Table 1). The percentages of malignant lesions in the training and test datasets were 74.2% and 71.4%, respectively. There were no significant differences in lesion size or endobronchial ultrasound visualization. In malignant lesions, there was no significant difference in stage (according to the eighth edition of the TNM classification) between the training and test datasets (Table 2).

**Table 1:** Clinicopathological characteristics.

Diagnosis	Training dataset (N = 171)	Test dataset (N = 42)
Malignant lesions (%)	N = 127	N = 30
Adenocarcinoma	76 (60)	16 (53)
Squamous cell carcinoma	41 (32)	9 (30)
Small-cell lung cancer	10 (8)	5 (17)
Benign lesions (%)	N = 44	N = 12
Infectious diseases	19 (43)	2 (16)
Organizing pneumonia	4 (9)	1 (8)
Sarcoidosis/Amyloidoma/Fibrosis/Hamartoma	3 (7)/2 (4.5)/2 (4.5)/0	0/0/0/1(8)
Benign (spontaneously disappear)	14 (32)	8 (68)

**Table 2:** Patients’ characteristics.

	Training dataset (N = 171)	Test dataset (N = 42)	*P*-value
Age, year, mean	72.7 ± 10.8	74.8 ± 12.6	0.072
Sex, female (%)/male (%)	57 (33)/114 (77)	12 (29)/30 (71)	0.5873
Lesion size, cm, mean	3.5 ± 2.1	3.1 ± 1.9	0.1856
Endobronchial ultrasound visualization, Adjacent to (%)/Within (%)	35 (20)/136 (80)	12 (29)/30 (71)	0.2992
Stage (%)			0.8394
IA-B	51 (40)	12 (40)	
IIA-B	9 (7)	1 (3)	
IIIA-C	20 (16)	4 (13)	
IVA-B	47 (37)	13 (44)	

### Model performance

**Figure 3:**
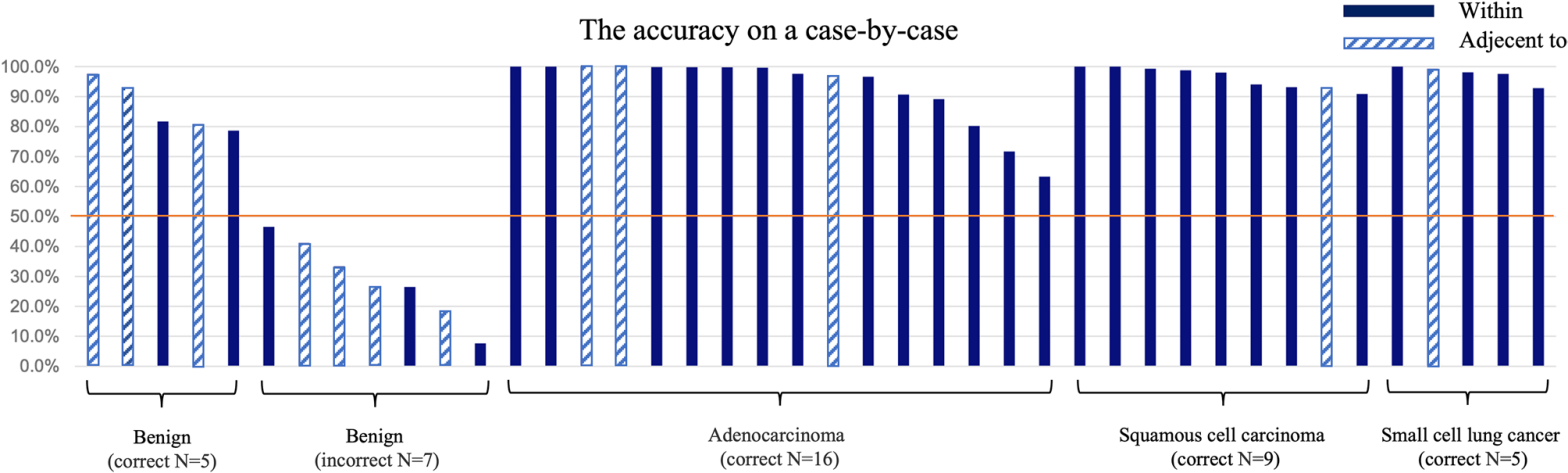
Accuracy for each case. For each case, when the ratio of images estimated to be correct was 50% or more, it was judged to be correct. When the endobronchial ultrasound visualization was adjacent to case, the graph showed a sprite pattern.

In the test dataset, 26,674 EBUS images of 42 peripheral pulmonary lesions were analyzed. The CNN-CAD system to differentiate malignant lesions (19,075 images) from benign lesions (7,599 images) showed an accuracy, sensitivity, specificity, PPV, and NPV of 83.4% (95% CI: 83.0–83.9%), 95.3% (95% CI: 95.0–95.6%), 53.4% (95% CI: 52.3–54.6%), 83.8% (95% CI: 83.3–84.3%), and 82.0% (95% CI: 80.9–83.0%), respectively (Table 3). For each case, when the ratio of images estimated to be correct was 50% or more, the result was judged to be correct. Even if the positional relationship between the probe and the lesion was “adjacent to,” malignancy could be diagnosed (Fig. 3). In the 42 test cases (30 cases of malignant and 12 cases of benign lesions), the accuracy, sensitivity, specificity, PPV, and NPV of the CNN-CAD system in differentiating lung cancer patients from those with benign lesions were 83.3% (95% CI: 68.6–93.0%), 100% (95% CI: 83.3–100%), 41.7% (95% CI: 15.2–72.3%), 81.1% (95% CI: 64.8–92.0%), and 100% (95% CI: 35.9–100%), respectively (Table 3). As with the CNN-CAD system, for the four bronchoscopists, if more than 50% reached the correct decision, the result was judged to be correct. On comparison, the accuracy of the CNN-CAD system was found to be higher than that of the four bronchoscopists (*p* = 0.0433) (Table 4).

**Table 3:** Diagnostic performance of the convolutional neural network computer-aided detection system compared to bronchoscopist.

	CNN-CAD	CNN-CAD	Bronchoscopist				Total (N = 4)
	(All images)	(Each case)	Expert 1	Expert 2	Trainee 1	Trainee 2	
Accuracy, % (95% CI)	83.4 (83.0–83.9)	83.3 (68.6–93.0)	73.8 (58.0–86.1)	66.7 (50.5–80.4)	57.1 (41.0–72.3)	76.2 (60.5–87.9)	68.5 (60.8–75.4)
Sensitivity, % (95% CI)	95.3 (95.0–95.6)	100 (83.3–100)	80 (61.4–92.3)	83.3 (65.3–94.4)	63.3 (43.9–80.1)	93.3 (77.9–99.2)	80.0 (71.7–86.7)
Specificity, % (95% CI)	53.4 (52.3–54.6)	41.7 (15.2–72.3)	58.3 (27.7–84.8)	25 (5.5–57.2)	41.7 (15.2–72.3)	33.3 (9.9–65.1)	39.6 (25.8–54.7)
PPV, % (95% CI)	83.8 (83.3–84.3)	81.1 (64.8–92.0)	82.8 (64.2–94.2)	73.5 (55.6–87.1)	73.1 (52.2–88.4)	77.8 (60.8–89.9)	76.8 (68.4–83.9)
NPV, % (95% CI)	82.0 (80.9–83.0)	100 (35.9–100)	53.8 (25.1–80.8)	37.5 (8.5–75.5)	31.2 (11.0–58.7)	66.7 (22.3–95.7)	44.2 (29.1–60.1)

*CNN-CAD* convolutional neural network computer-aided detection, *PPV* positive predictive value, *NPV* negative predictive value.

**Table 4:** Comparison of accuracy between the CNN-CAD model and four bronchoscopists.

		Bronchoscopists (total N = 4)		Accuracy
		Correct	Incorrect	*P*-value
CNN-CAD (each case)	Correct	25	10	0.0433
	Incorrect	2	5	

*CNN-CAD* convolutional neural network computer-aided detection.

**Figure 4:**
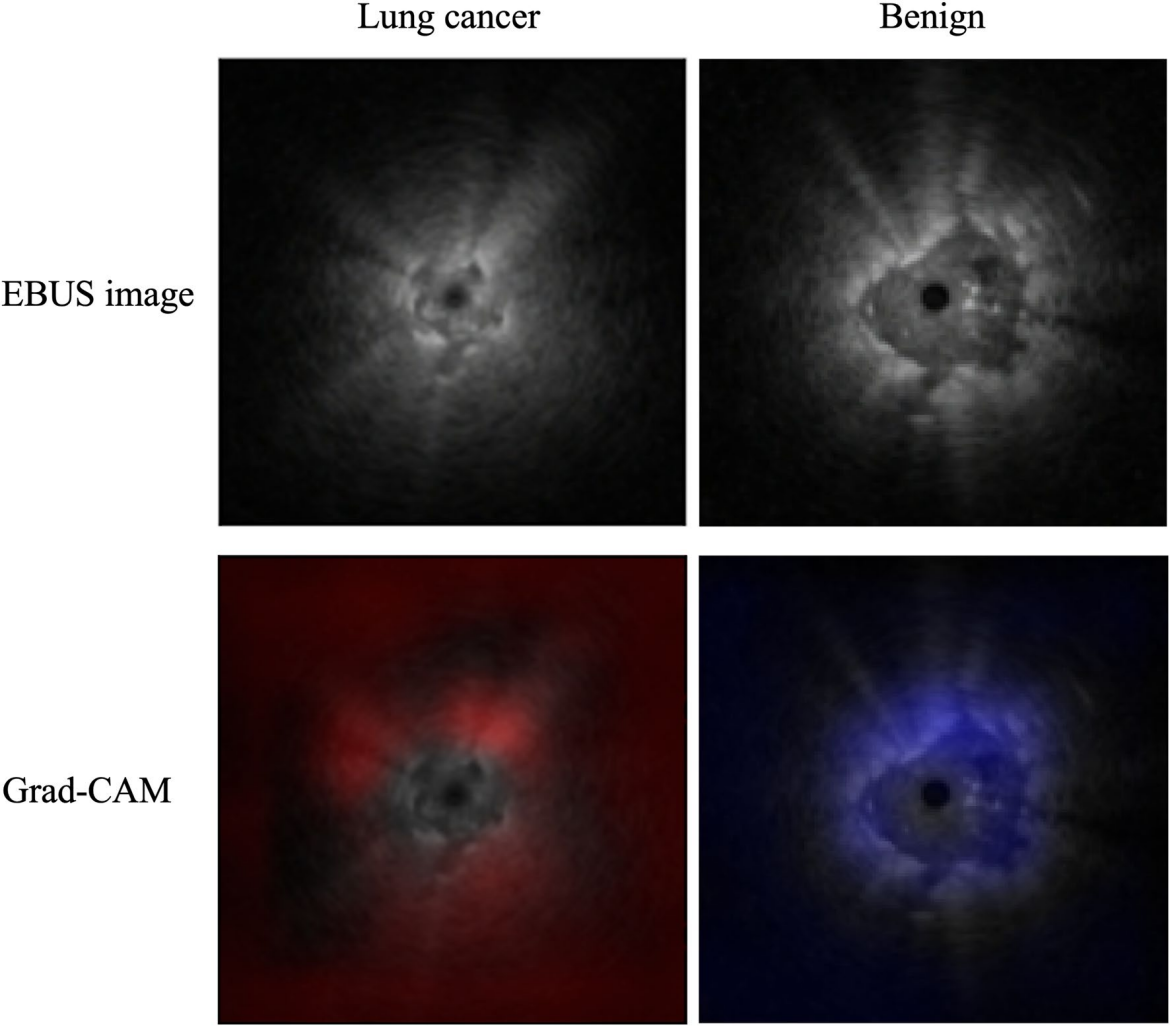
Visualization techniques; Gradient-weighted Class Activation Mapping. Areas suspected of being malignant are shown in red and areas suspected to be benign in blue.

Visualization was performed using gradient-weighted class activation mapping. The regions of interest (malignant lesions) by CNN were visualized in red, and the regions of interest (benign lesions) were visualized in blue (Fig. 4).

## Discussion

Our CNN-CAD system differentiated lung cancer from benign lung lesions with an accuracy of 83.4% in the independent test dataset. Furthermore, in a case-by-case analysis, the CNN-CAD system achieved a sensitivity of 100%. To the best of our knowledge, this is the first study to report the efficacy of the CNN-CAD system in distinguishing lung cancer from EBUS images.

The findings obtained by EBUS show the positional relationship between the ultrasonic probe and the lesion, and these positions are roughly divided into three patterns: within (lesion visualized all around), adjacent to (visualized adjacent to the core lesion), and invisible (lesion not visualized at all). The diagnosis rate differs depending on this positional relationship, and if the tumor cannot be physically reached by the biopsy device, tissue cannot be collected and the diagnosis rate drops to approximately 60% or less^1–3^. This study included adjacent to cases for both learning and testing datasets (Fig. 2). An accuracy of 83.4% was demonstrated, which points towards a room for improvement, but the technique may be useful in the cases where the EBUS probe is adjacent to the lesion.

Regarding the use of ultrasound images in bronchoscopy, it has been reported that convex probe endobronchial ultrasound sonographic images are useful. In a previous study^10^, a deep learning model was used to determine whether the mediastinal lymph nodes were benign or malignant. The accuracy was reported as 88.57%. Use of AI enables real-time diagnosis of a lesion, and if benign and malignant lesions can be distinguished based on the ultrasonic images, unnecessary biopsy can be avoided. We believe that diagnostic assistance using AI is useful not only for improving the accuracy of diagnosis but also for maintaining safety.

Methods for distinguishing benign and malignant lesions by using EBUS, which were based on the internal structure of the lesion, have been reported in the literature. The focus was on internal echo, bronchial and vascular patency, and morphology of the hyperechoic region^4^. In this study, the visualization of CNN-CAD system suggests that AI pays attention not only to the internal structure but also to the edges. AI may reflect differences that are undetectable to the human eye, such as echo attenuation.

One limitation of this study is that it was an observational study conducted in a single facility. However, virtual bronchoscopic navigation^11^ or electromagnetic navigation bronchoscopy systems^12,13^ have emerged as a means of supporting biopsy-based diagnosis of peripheral pulmonary lesions. The existing technical differences between the various facilities are being equalized by using an image-guided system.

Another limitation of this study was the lack of data on benign diseases, which reduced the specificity of the obtained results. However, due to the nature of bronchoscopy itself, which deals mainly with malignancies, sensitivity takes precedence over specificity. As a multicenter study, it is also necessary to collect data on benign diseases. In the future, we aim to conduct studies to determine whether real-time evaluation of EBUS data during bronchoscopy can contribute to the diagnostic accuracy.

In conclusion, we can state that use of CNN-CAD system for diagnosing peripheral pulmonary lesions aids in the accurate diagnosis of lung cancer.

## Data Availability

The datasets generated and analyzed during the current study are not publicly available due to the waiver of the requirement of consent from patients, but are available from the corresponding author on reasonable request. The data provided will be de-identified, not raw data.
